# Supplementary figures and images for: Traveling waves in the human visual cortex: An MEG-EEG model-based approach
Source: PLoS Comput Biol. 2025 Apr 17;21(4):e1013007. doi: 10.1371/journal.pcbi.1013007 (PMC12037073; doi:10.1371/journal.pcbi.1013007)

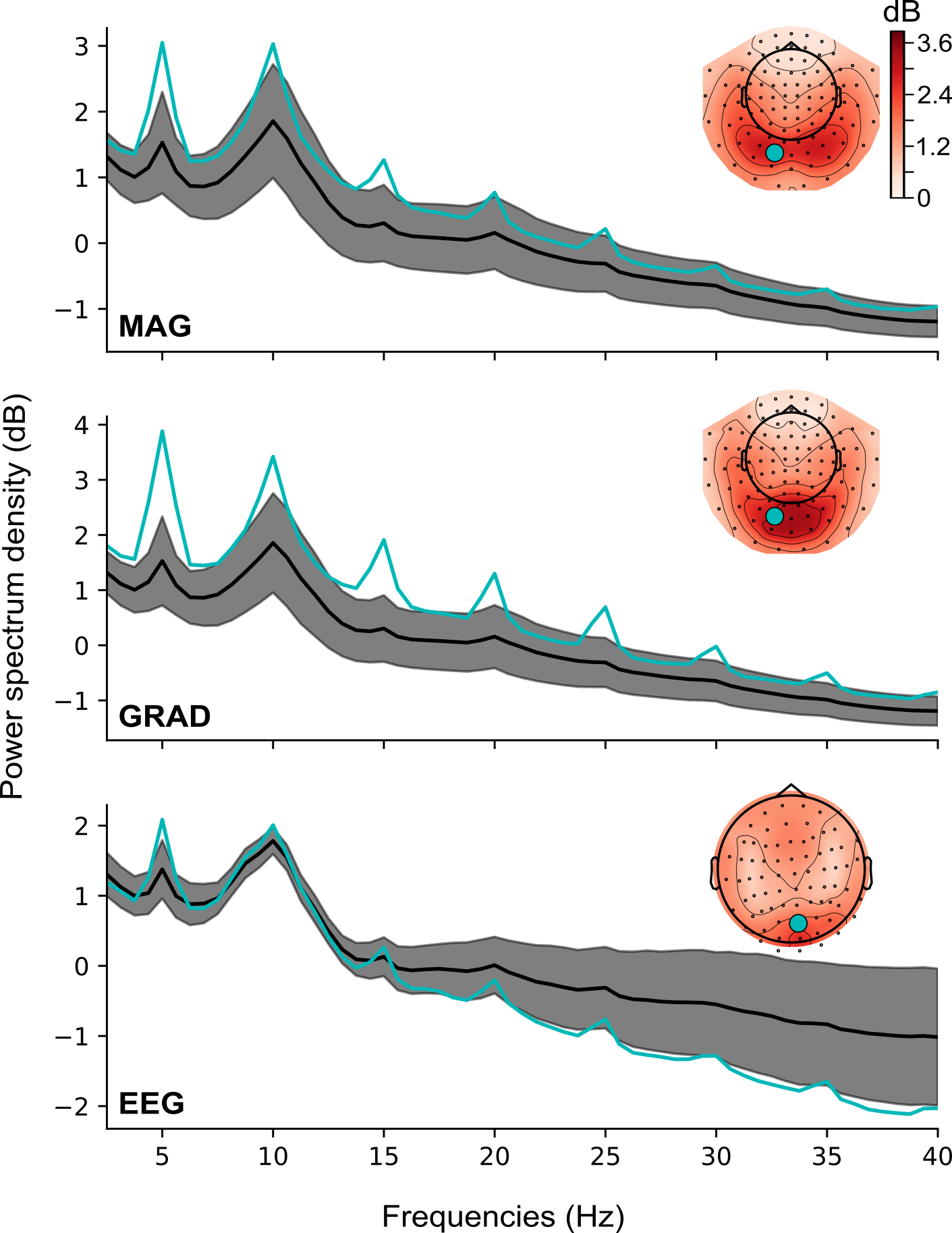

Supplement: S1 Fig — The group-averaged PSD is averaged across sensors (black line: mean, shaded area: SEM) and plotted for a selected sensor (blue). The 5-Hz component and its harmonics are clearly visible. The inset topomaps show the PSD at 5 Hz for each sensor type. (TIFF) [file pcbi.1013007.s001.tiff]

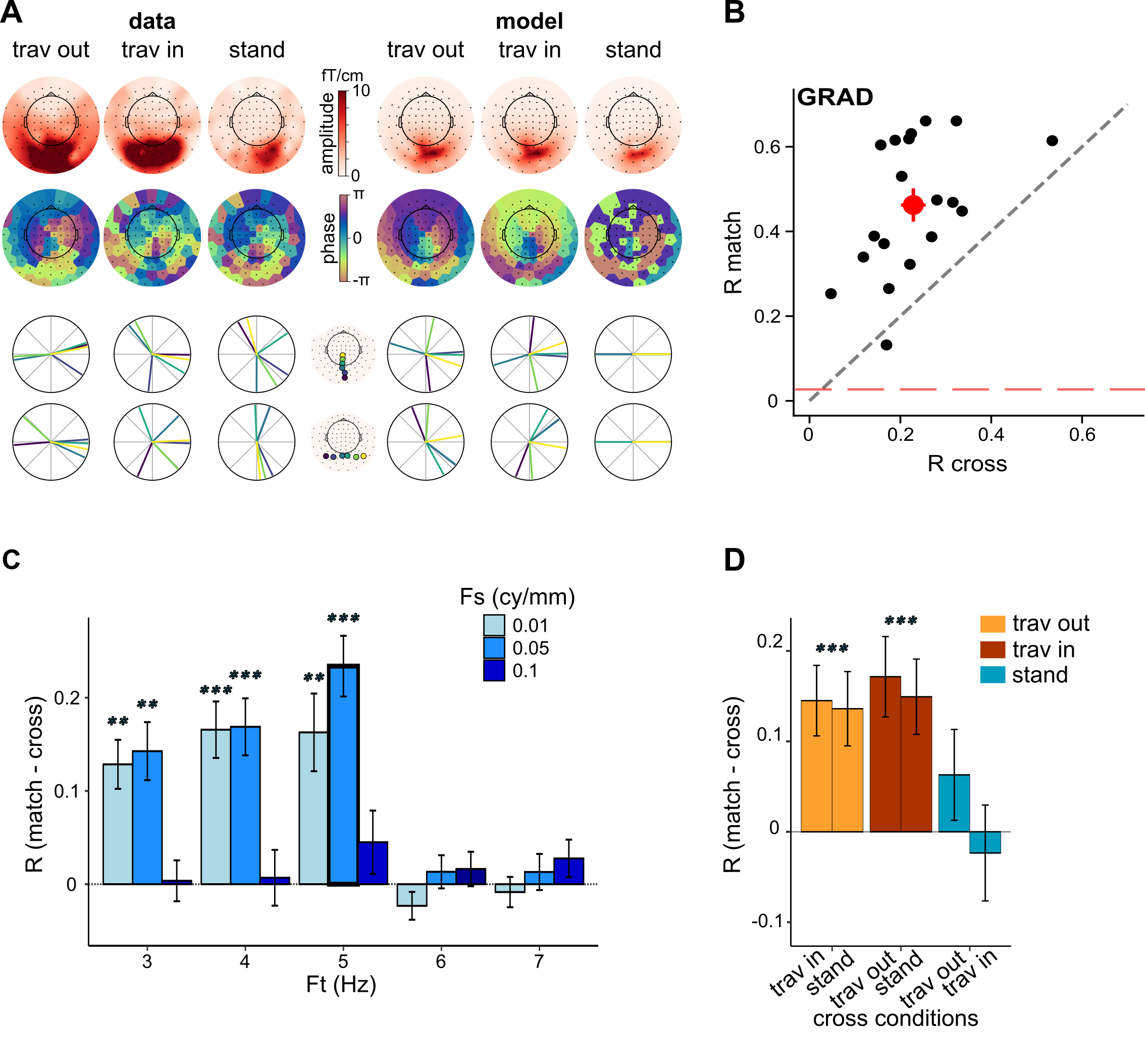

Supplement: S2 Fig — Same legend as for Figs 4 and 5. (TIFF) [file pcbi.1013007.s002.tiff]

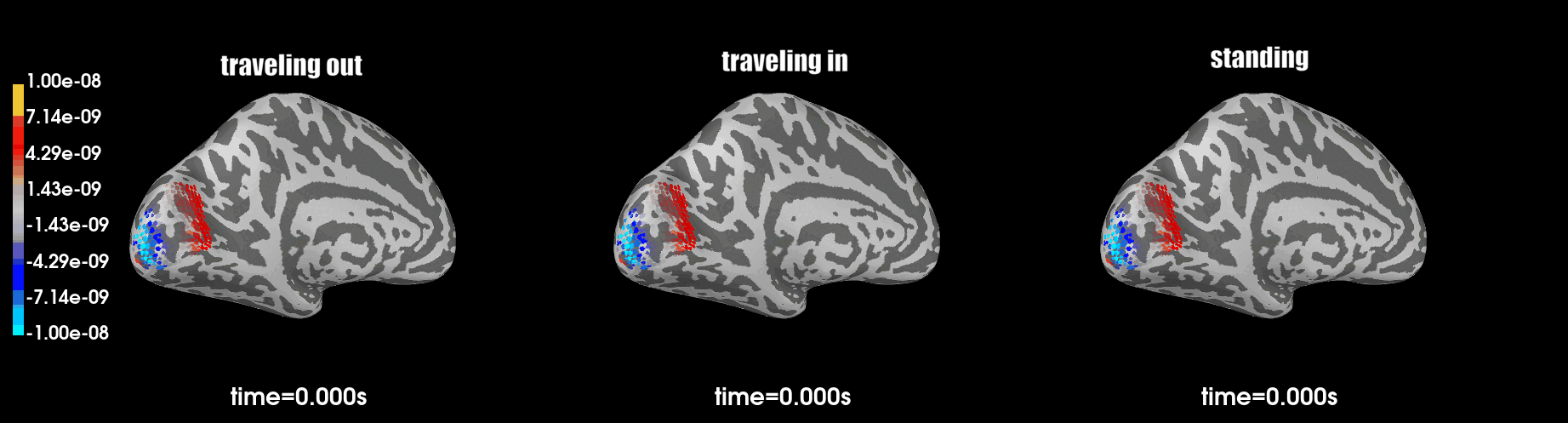

Supplement: S1 Video — (GIF) [file pcbi.1013007.s003.gif]

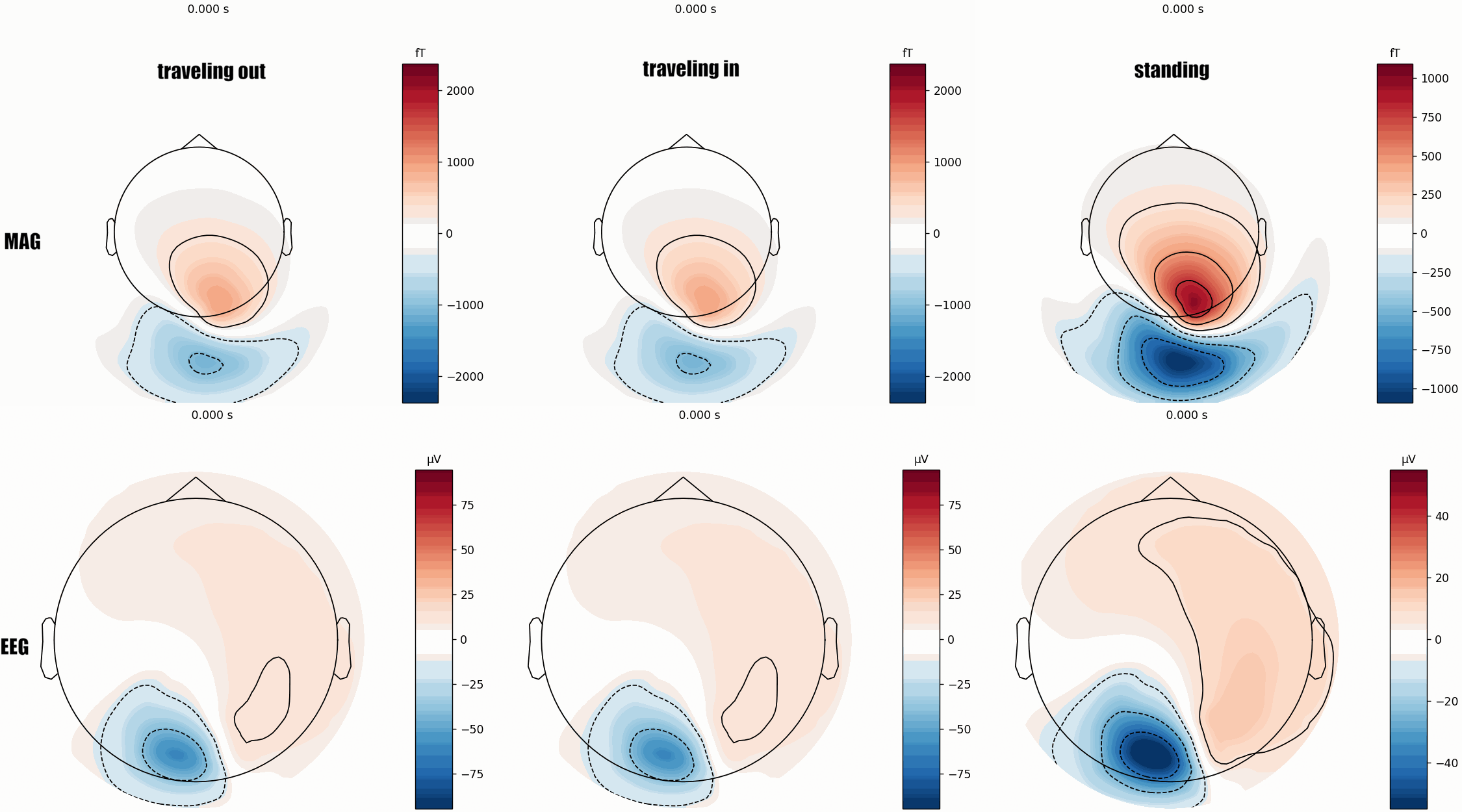

Supplement: S2 Video — (GIF) [file pcbi.1013007.s004.gif]
