# Supplementary material for: Traveling waves in the human visual cortex: An MEG-EEG model-based approach
Source: PLoS Comput Biol. 2025 Apr 17;21(4):e1013007. doi: 10.1371/journal.pcbi.1013007 (PMC12037073; doi:10.1371/journal.pcbi.1013007)
Supplement: Attachment — Submitted filename: Grabot_etal_Response to Reviewers.pdf [file pcbi.1013007.s006.pdf]

February 7th, 2025

Dear PLOS Computational Biology Editors,

We thank you and the reviewers for evaluating our manuscript and are happy of the interest you expressed.

Below, we indicate how and where in the manuscript (page and line numbers) we have addressed each and all of the reviewer's comments [new or revised text is in blue]. We have added a figure of the experimental procedure, given measures of effect size and models' discriminability, and expanded the Discussion section, notably by giving concrete examples for applications of our model, practical recommendations and by detailing potential model extensions. As a result of the useful feedback, our manuscript is stronger and clearer.

We hope you find the revised manuscript suitable for publication in PLOS Computational Biology and look forward to hearing from you.

Best regards,

Laetitia Grabot, Garance Merholz, Jonathan Winawer, David Heeger and Laura Dugué

Reviewer's Responses to Questions

Additional Editor Comments:

All three reviewers have deemed the work interesting, original and well-written. They have also made suggestions for minor improvements and for broadening the discussion that you will want to consider.
Thank you for the positive feedback. We have addressed the reviewers' suggestions for improvements and expanded the discussion as recommended.

Reviewer #1:

It is an interesting study; the reviewer has some comments.
Thank you for the positive feedback and the useful comments.

1. Please consider further exploring why the standing model does not show greater specificity. Alternatively, discuss in more detail the implications of standing stimuli potentially generating endogenous traveling waves.
We have explored further a potential explanation for the lack of specificity of the standing model that might come from methodological considerations related to the degree of discriminability between the models. We have added this analysis in the **Results** section [**page 8, lines 33-47**].
We have also expanded the **Discussion** [**page 10, lines 7-22**] regarding standing stimuli generating endogenous traveling waves, by mentioning that traveling waves might be endogenously generated by other visual areas in addition to V1. This would affect the specificity of the standing model.

2. The study highlights differences in the specificity and predicted topographies for MEG and EEG, with EEG predictions showing less granularity and specificity. Please elaborate on the practical implications of these differences.
We have expanded the **Discussion** [**page 11, lines 1-4**] as suggested.

3. The study is currently limited to simulating activity in V1. Provide a brief outline of how the model could be expanded to include multiple areas.
The focus of our study is indeed on V1 as a proof-of-principle, but it is important to later extend the model to include multiple areas. We have expanded the **Discussion** [**page 11, lines 11-18**] as suggested.

4. The study briefly mentions that the tool could test hypotheses about neural oscillation propagation and the generation of MEG-EEG signals. Please strengthen this point by providing concrete examples of such hypotheses and how this tool could be applied.
We have strengthened this point in the **Discussion** [**page 11, lines 20-36**] as suggested.

5. The model explains over 44% of the variance in the data, which is promising but leaves a significant portion unexplained. Please discuss the potential sources of unexplained variance.
We have first emphasized in the **Results** section [**page 5, lines 21-23**] that simulating neural activity in V1 alone affects all sensors and explains a large amount of the data (~44%).

As pointed out by the reviewer, however, we now must develop a more encompassing model. We have added further **Discussion** [**page 11, lines 9-11**] on the potential sources of the currently unexplained variance.

6. Please expand on the observed differences between MEG and EEG predictions in the Discussion section. Specifically, consider elaborating on the practical implications of these differences for integrating MEG and EEG data in future studies.
We believe that this comment is the same as the reviewer's Point #2. Please see our response above.

Reviewer #2:

In the present work, Grabot and colleagues aim at developing a model-based neuroimaging approach to assess traveling waves in non-invasive recordings. They compared the model's performances to MEG-EEG signals obtained during controlled experimental conditions. The authors were able to explain 44% of the variance of data.
The methodology is rigorous and the results well presented, with useful and detailed figures. I have only a few minor concerns that I would ask the authors to address to improve the manuscript's clarity.
Thank you for the positive feedback and the useful comments.

INTORDUCTION
1- Before starting describing the two-part model, I would add a brief description of the EEG and MEG methodologies, especially focusing on the fact that spacial discrimination is not their main strenght.
We have strengthened this point in the **Introduction** [**page 3, lines 33-37**].

2-I would also suggest to better define "standing" and "travelling" waves.
We have clarified these terms in the **Introduction** [**page 3, lines 49-51**].

RESULTS
3- The authors say that they can explain around 44% of the variance of the data: I would add a comparison with other models or a sort of comparator to give an effect size of predictive performance (mild-moderate-great).
We calculated an effect size based on the comparison of the correlation coefficients using the matched model versus the crossed models. We have added this information in the **Methods** [**page 16, lines 13-16**] and **Results** sections [**page 6, lines 20 and 22**].

DISCUSSION
4- Ath the end of the discussion session, I would reprase the conclusive sentence "This approach aligns with the advocated need for an integrated view of brain functioning across time and space to understand the multiscale basis of cognition". Given that the authors claim it's the first work of the kind, I would not overstate the implications of their novel approach.
We have rephrased the conclusive sentence in the **Discussion** [**page 11, lines 36-38**]. Additionally, in response to Reviewer #1's Point #4, we have provided concrete examples of hypotheses our tool can test, which clarifies and strengthens the concluding sentence.

Reviewer #3:

This is a very intriguing, novel, well-designed, and well-written work. I have only a few minor comments.
Thank you for the positive feedback and the useful comments.

1. Please provide full information about the funding: Initials of the authors who received each

award; URL of each funder website; Did the sponsors or funders play any role in the study design, data collection and analysis, decision to publish, or preparation of the manuscript?
We have addressed this point [**page 16, lines 35-37**].

2. Please indicate a possible significance of the results in cognition. The authors only briefly mention this matter in one sentence. I'd like the Authors to expand this fragment.
Reviewer #1 had a similar comment (see Point #4). We have expanded this section in the **Discussion** [**page 11, lines 20-36**].

3. Please provide explanations of the abbreviations used in the figures. E.g. in fig. 1.: 'VM' and 'HM'.
We made the abbreviations in **Figures 1, 2 and 4** explicit.

Experimental procedure
4. Could you please add the mean total time of the experimental session?
We have added this information in the **Methods** section [**page 14, lines 2-3**].

5. A figure presenting the experimental protocol would also help in visualizing the details of the experimental procedure.
We have added the experimental protocol in **Panel C** of **Figure 1**.

6. Since the color change to maintain participants' attention occurred during the M/EEG epoch - were these trials excluded from the analysis?
We have clarified in the **Methods** section [**page 14, lines 12-14**] that because of the low number of trials in which a color change could occur (20 out of 640 trials), their random timing, and their brief duration, potential effects on the MEG-EEG signals were minimized during trial averaging. All trials were therefore included in analyses.

7. MEG-EEG pre-processing
How were the blinks during the stimulus presentation handled? Usually, before the ICA, the stimuli during which an eye blink occurred are identified, and then these trials are excluded from the analysis to prevent analyzing trials where the participants did not see the stimulus.
We agree that this procedure is classically used when very brief stimuli are presented. Here, the stimuli lasted 2s. So even if in the case of a blink or a saccade during stimulus presentation, only a small portion of the trial was missed. We therefore did not exclude trials based on blink or saccade but removed them with the ICA procedure based on the EOGs. We have clarified the **Methods** section accordingly [**page 14, lines 48-49**].

8. Comparison between predicted and measured data
I think the information provided in this section (or at least the first part) should be moved to the 'MEG-EEG pre-processing' section.
We have moved the information [**page 15, lines 4-11**].

9. Results
Some of the information is repeated after other sections.
We are not entirely certain which specific information the Reviewer is referring to. However, if we repeated details already presented in the Methods section, it was done intentionally to ensure the Results section is as self-contained and comprehensible as possible.
